# Retrospective evaluation of 22 dogs with leptospirosis treated with extracorporeal renal replacement therapies (2018‐2021)

**DOI:** 10.1111/jvim.16998

**Published:** 2024-02-09

**Authors:** Antonia Da Fonseca Ioannou, Carolyn Tai, Mary Anna Labato, Emmanuelle M. Butty

**Affiliations:** ^1^ Department of Clinical Sciences Tufts University, Cummings School of Veterinary Medicine, Foster Hospital for Small Animals North Grafton Massachusetts USA

**Keywords:** acute kidney injury, anuria, azotemia, canine, continuous renal replacement therapy, hemoperfusion, intermittent hemodialysis, *Leptospira*, oliguria

## Abstract

**Background:**

Outcomes of dogs with acute kidney injury secondary to leptospirosis (AKI‐L) treated using renal replacement therapies (RRT) are poorly characterized.

**Hypothesis/Objectives:**

Describe survival to discharge, short (≤30 days) and long‐term (≥6 months) outcomes of AKI‐L dogs receiving RRT and determine if there is a significant difference in maximum blood urea nitrogen (maxBUN), maximum creatinine (maxCr), maximum bilirubin (maxBili) and the number of body systems affected between survivors and non‐survivors.

**Animals:**

Twenty‐two client‐owned dogs with AKI‐L receiving RRT.

**Methods:**

Retrospective medical record review of dogs with AKI‐L that received RRT between 2018 and 2021.

**Results:**

Sixteen of 22 (73%) dogs survived to discharge. Of the survivors, 13 (81%) were alive >30 days from discharge and 12 (75%) were alive at 6 months from discharge. Factors significantly higher in non‐survivors included number of body systems affected (survivors: 1 (19%), 2 (50%), 3 (25%) and 4 (6%) vs non‐survivors: 3 (33.3%), and 4 (66.7%); *P* = .01) and median maxBili (survivors: 1.9 mg/dL; range, 0.1‐41.6 vs non‐survivors: 21.0 mg/dL; range, 12.3‐38.9; *P* = .02). There was no significant difference in median maxBUN (survivors: 153.0 mg/dL; range, 67‐257 vs non‐survivors: 185.5 mg/dL; range, 102‐218; *P* = .44) and median maxCr (survivors: 9.8 mg/dL; range, 6.2‐15.9 vs non‐survivors: 9.8 mg/dL; range, 8.4‐13.5; *P* = .69) between survivors and non‐survivors.

**Conclusions and Clinical Importance:**

Regardless of azotemia severity, dogs with AKI‐L receiving RRT have a good survival rate to discharge. The number of body systems affected and hyperbilirubinemia might be associated with worse outcomes.

**Abbreviations**
AKI: acute kidney injury
AKI‐L: acute kidney injury secondary to leptospirosis
IHD: intermittent hemodialysis
maxBUN: maximum serum blood urea nitrogen
maxCr: maximum serum creatinine
maxBili: maximum serum bilirubin
minPLT: minimum platelet count
RRT: renal replacement therapy
CRRT: continuous renal replacement therapy

## 1 INTRODUCTION

Leptospirosis is a widespread, worldwide zoonotic disease caused by spirochetes of the genus Leptospira.^1^ In dogs, leptospirosis results in inflammatory, multisystemic disease, predominantly affecting the kidneys and liver.^2^, ^3^ Body systems that might be affected include the lungs, vascular endothelium, coagulation system, heart and eyes.^2^, ^3^, ^4^ Survival depends on clinical sign severity and secondary complications.^3^, ^5^ In earlier studies mainly describing cases treated with medical management, severe azotemia was associated with a poor prognosis.^5^, ^6^, ^7^

During the disease's acute phase, dogs typically are presented with an AKI secondary to acute interstitial nephritis.^8^ Thirty percent of dogs with acute leptospirosis develop oliguria or anuria.^4^ These dogs might die before they reach the recovery stage of kidney function and therefore extracorporeal renal replacement therapy (RRT) is recommended.^9^ This therapy can remove uremic toxins, correct fluid and electrolyte disorders and restore acid‐base balance until the recovery phase begins.^9^ A survival rate of 86% is reported in dogs with an AKI‐L that received treatment with intermittent hemodialysis (IHD).^5^ Since the publication of that report, there has been expanded veterinary experience and an increased availability of RRT.^2^, ^3^, ^10^, ^11^, ^12^ There is a need for updated information on the survival of dogs with AKI‐L when RRT is available, and on factors that might affect their prognosis.

The aim of this retrospective study was to describe the survival to discharge, short‐term (≤30 days from discharge) and long‐term (≥6 months from discharge) outcome in dogs with AKI‐L requiring RRT. A secondary aim was to determine if maximum blood urea nitrogen (maxBUN), maximum serum creatinine (maxCr), maximum bilirubin (maxBili) and the number of body systems affected might be associated with survival to discharge.

## 2 MATERIALS AND METHODS

The medical records of dogs diagnosed with leptospirosis and treated with RRT between January 2018 and December 2021 at *Tufts Cummings School of Veterinary Medicine (TCSVM)* were retrospectively reviewed.

### 2.1 Inclusion and exclusion criteria

Inclusion criteria included a complete medical record, a confirmed diagnosis of leptospirosis and treatment with at least 1 session of RRT.

Criteria for leptospirosis diagnosis included a single micro agglutination test (MAT) titer >1:800 for nonvaccine serovars or >1:1600 for vaccine serovars, a greater or equal than 4‐fold increase in convalescent titers performed 14 days after original titer, or a positive urine or blood polymerase chain reaction (PCR) test. Exclusion criteria included incomplete medical records, dogs not diagnosed with leptospirosis using the criteria above, and dogs with AKI‐L not requiring RRT.

### 2.2 Diagnostics and hospitalization

The extracorporeal therapy database at *TCSVM* was screened for dogs who received RRT and were tested for leptospirosis between 2018 and 2021. Dogs with a confirmed leptospirosis diagnosis had medical records at *TCSVM* and referring veterinarian records reviewed for signalment, presenting clinical signs, clinical parameters (heart rate, respiratory rate, temperature, weight), hydration status and urine production.

Fluid overload was clinically assessed based on >10% weight gain from euhydrated baseline, chemosis, peripheral edema, ascites, pleural effusion, pulmonary edema or serous nasal discharge.^13^ Urine output (UOP) was classified as polyuria (>2 mL/kg/h), oliguria (<1 mL/kg/h) and anuria (no urine production >6 hours)^14^ and was measured before RRT by either referring veterinarians or via an indwelling urinary catheter at our institution. Vital parameters were evaluated to assess for persistent tachypnea (>40 breaths per minute) or dyspnea. Dogs that required transfusions were first blood typed then transfused with type‐specific blood. Dogs that had received a transfusion >72 hours prior had a major cross match performed and were transfused with a compatible blood product.

Complete blood count (CBC), serum biochemistry and urinalysis within 24 hours of RRT initiation were reviewed. Increased serum creatinine (sCr) was defined as sCr ≥1.6 mg/dL, increased serum BUN as BUN ≥30 mg/dL and AKI was defined based on IRIS AKI grading system.^14^ Clinical pathology evaluation performed during hospitalization was evaluated to determine the maxCr, maxBUN, maxBili and minimum platelet count (minPLT). Serum biochemistries and CBCs were performed in the clinical pathology laboratory at *TCSVM*, and blood smears were reviewed by a board‐certified clinical pathologist if abnormalities were present.

Additional testing performed during hospitalization included urine culture and sensitivity, coagulation testing before RRT. Coagulation testing included partial prothrombin time (PT) and thromboplastin time (aPTT) (Stago STart, Diagnostica Stago, Inc, Pasippany, New Jersey) and thromboelastography (TEG) (TEG 500 Thromboelastograph, Haemonetics Corp, Braintree, Massachusetts). Additional testing included heartworm and tick‐borne disease testing (SNAP 4DX IDEXX), and imaging (thoracic radiographs, abdominal ultrasound, echocardiography). All imaging studies were reviewed by board‐certified radiologists and echocardiograms by board‐certified cardiologists.

Leptospirosis testing methodologies and results were evaluated. Testing included point of care screening tests (WITNESS Lepto Rapid Test Zoetis, SNAP Lepto IDEXX), acute and convalescent MAT, and PCR on blood and urine. Microscopic agglutination test was performed according to World Health Organization and International Leptospirosis Society standards^15^ and measured serum antibodies against *Leptospira interrogans* serovar Autumnalis, Bratislava, Canicola, Icterhaemorrhagiae, Pomona and *Leptospira kirschneri* serovar Grippotyphosa. The urine or ethylenediamine tetra‐acetate anticoagulated blood PCR was performed according to World Organization for Animal Health (OIE) standards.^16^ A confirmatory diagnosis was made based on MAT and/or PCR testing.

### 2.3 Extracorporeal renal replacement therapy

Dogs were considered RRT candidates if they had a documented AKI with 1 or several of: inadequate urine production, hyperkalemia, fluid overload or progressive azotemia that was unresponsive to medical management.

All dogs had a size‐appropriate (range, 8 Fr × 12 cm to 14 Fr × 30 cm) double‐lumen temporary hemodialysis catheter placed in an external jugular vein using the modified Seldinger technique while under deep sedation or intubated under anesthesia. Intermittent hemodialysis sessions (IHD) were performed on a Fresenius 2008T platform (Fresenius Medical Care, Bad Homburg, Germany). A hybrid, intermittent version of diffusive and convective treatment using a continuous renal replacement therapy (CRRT) platform was performed on the Prismaflex platform (Gambro Lundia AB, Lund, Sweden) if the Fresenius 2008T platform was unavailable. The dialyzer was chosen based on patient size, degree of azotemia and priming volume. Priming was performed with 0.9% NaCl as the priming solution. For our most recent patient, a cytokine reduction filter, VetResQ (CytoSorbents, Inc, Monmouth Junction, New Jersey) was added pre‐dialyzer in series to the IHD circuit. All dogs received systemic anticoagulation with unfractionated heparin (UFH) or regional citrate anticoagulation (RCA). Dogs anticoagulated with UFH were monitored by activated clotting time (ACT) (ACT Plus Automated Coagulation Timer System, Medtronic, Minneapolis, Minnesota) to maintain the ACT within an acceptable range of 180 to 200 seconds. Dogs with a clinically relevant thrombocytopenia (<60 k/μL), were anticoagulated with RCA due to the bleeding risk associated with systemic anticoagulation. In accordance with a previously published protocol,^17^ 4% trisodium citrate was added to the circuit as the blood was withdrawn from the dialysis catheter's arterial port to lower the iCa in the extracorporeal circuit to a target range of 0.2 to 0.4 mmol/L. Just before the blood was returned to the patient, 10% calcium gluconate was added via the dialysis catheter's venous port to target an iCa of 0.8 to 1.2 mmol/L in the patient.

The dialysis prescription was adjusted to the needs of individual patients, according to published protocols.^18^ The target urea reduction ratio (URR) was determined based on the serum BUN before RRT and on the number of treatments in accordance with previously established safe URR targets.^18^ Ultrafiltration was performed in fluid‐overloaded dogs and ultrafiltration rate was based on the dog's clinical status. Monitoring and adjustments of ultrafiltration were based on the percent change in the dog's intravascular blood volume displayed on the Crit‐Line III monitor (Fresenius Medical Care, Waltham, Massachusetts). All dogs were monitored with a 6‐lead EKG, temperature probe and blood pressure monitoring.

Session records were reviewed for complications during individual RRT sessions. Complications were characterized as not requiring intervention, requiring emergent intervention and requiring urgent session termination. Dialysis disequilibrium was defined as a central nervous system disturbance caused by a rapid decrease in serum osmolality due to a highly effective RRT session.^19^

### 2.4 Body systems affected

Dogs were grouped by the number of body systems affected based on the target organs of leptospirosis.^2^, ^3^ Body systems affected were determined based on CBC, serum biochemical profiles, urinalysis, coagulation testing, imaging and concurrent clinical signs. Kidney involvement was defined according to the IRIS AKI grading scheme.^14^ Hepatic involvement was defined as elevations in 2 or more serum liver enzyme activity, with or without hyperbilirubinemia. Dogs with ultrasonographic pancreatic or gallbladder abnormalities without increased liver enzyme activity were not considered to have hepatic involvement. Pulmonary involvement was defined as persistent tachypnea and dyspnea with either evidence of an alveolar or interstitial pattern on thoracic radiographs or evidence of lung disease on necropsy. Coagulation system involvement was defined as abnormal results of coagulation testing before RRT, clinically relevant thrombocytopenia (minPLT <60 k/μL) or clinical evidence of thrombi or bleeding tendencies (epistaxis, petechial hemorrhages).

### 2.5 Outcomes

Survival to discharge was defined as dogs alive at hospital discharge. Serum creatinine at discharge was recorded for surviving dogs and before death for non‐surviving dogs. Assessment of short‐term survival (≤30 days) and long‐term (≥6 months) survival were performed by reviewing records at *TCSVM* and from referring veterinarians to determine whether a dog that survived to discharge was alive or dead at 30 days and at 6 months from discharge. Dogs alive after 6 months from discharge, were staged according to IRIS CKD Staging guidelines^20^ based on their most recent sCr.

### 2.6 Statistical analysis

Data were tested for normality using histograms. For continuous variables, median and ranges were computed. For noncontinuous variables, frequencies and percentages were reported. To determine if there were differences based on survival status, Wilcoxon rank sum tests and Fisher's exact test (for noncontinuous variables) were employed due to low numbers of data points. Spearman correlation analysis was used to assess the association between maxBili and maxBUN and maxCr. All analyses were conducted using RStudio v.4.2.2^21^ and 0.05 was used as a level of significance.

## 3 RESULTS

### 3.1 Animals

Twenty‐four dogs received treatment with RRT for management of AKI‐L between January 2018 and December 2021. Two dogs without confirmed diagnosis of leptospirosis were excluded. The remaining 22 dogs were included in the study.

Fourteen dogs (64%) were males (8 castrated, 6 intact) and 8 dogs (36%) were females (7 spayed, 1 intact). The median age and weight on presentation was 5.1 years (range, 0.25‐11 years) and 14.0 kg (range, 6.4‐29.3 kg) respectively. Breeds represented included 4 mixed breeds, 3 Golden retrievers, 2 Labrador retrievers, 2 German shepherds and 1 of the following: Bulldog, Siberian husky, Pekingese, Border collie, Australian shepherd, Cairn terrier, Dachshund, Poodle, Rat terrier, West Highland White Terrier and Rhodesian Ridgeback.

### 3.2 Testing for leptospirosis

Leptospirosis testing performed included WITNESS Lepto Rapid Test (Zoetis) (n = 3) SNAP Lepto (IDEXX laboratories, Westbrook, Maine) (5), acute MAT (20), convalescent MAT (7) and PCR (15). The serovars with the highest titers on MAT included Bratislava (9/22, 41%, 1 : 800‐1 : 51 200) and Gryppotyphosa (9/22, 41%, 1 : 800‐1 : 51 200). Other serovars included Icterohaemorrhagiae (6/22, 27%, 1 : 800‐1 : 6400), Autumnalis (5/22, 23%, 1 : 800‐12 800) and Pomona (4/22, 18%, 1 : 600‐1 : 51 200). No dogs had positive results for Canicola.

### 3.3 Clinical presentation

Twenty‐one dogs (95%) were referred for RRT. Before referral, 20 dogs (95%) were hospitalized at another hospital for a median of 2 days (range, 1‐4). Eighteen dogs (82%) had received antibiotic therapy before presentation. One dog had received treatment for hypertension with amlodipine.

Clinical signs on presentation included vomiting (20/22, 91%), anorexia (17/22, 77%), lethargy (13/22, 59%), diarrhea (8/22, 36%) and weakness (3/22, 14%). Physical examination findings included icterus (9/22, 41%), tachypnea or dyspnea (4/22, 18%) and peripheral edema (7/22, 32%). Five dogs (23%) were estimated to be dehydrated and 13 (59%) were assessed to be fluid overloaded. The median blood pressure on presentation was 155.8 mmHg (range, 113‐240 mmHg). On intake, 7 dogs (32%) were anuric, 8 (36%) were oliguric, 5 (23%) were polyuric and 2 (9%) had a normal UOP. One dog with a normal UOP became oliguric after intake.

### 3.4 Hospitalization and laboratory results

All dogs had a CBC, serum biochemistry and urinalysis performed (Table 1). Sixteen dogs (73%) were anemic and 8 required transfusions which included 1 or more of the following: whole blood (1), packed red blood cells (7) and plasma (3). All dogs were azotemic. The overall median maxBUN and maxCr were 159 mg/dL (range, 67‐257) and 9.8 mg/dL (range, 6.2‐15.9), respectively. The IRIS AKI grades included 12/22 (55%) dogs with Grade IV, and 10/22 (45%) with Grade V.

**TABLE 1:** Clinical‐pathological results of dogs with leptospirosis within 24 hours of first treatment with RRT.

Variable	Median (range)	Proportion (%) abnormal values	Reference range
White blood cell count (n = 22)	17.5 (10.5‐34.6)	16/22 (78%)	16/22 (78%)
Hematocrit (n = 22)	37.0 (23.0‐54.9)	16/22 (78%)	16/22 (78%)
Platelet (n = 20)	115 (46‐263)	15/20 (75%)	15/20 (75%)
Reticulocyte^a^ (n = 22)	27.8 (6.7‐78.4)	‐	‐
Neutrophil^a^ (n = 20)	14.505 (8.000‐27.656)	15/20 (75%)	15/20 (75%)
Lymphocyte^a^ (n = 22)	1.46 (0.38‐7.65)	7/22 (32%)	7/22 (32%)
Monocyte^a^ (n = 22)	1.10 (0.30‐2.62)	7/22 (32%)	7/22 (32%)
BUN (n = 22)	152 (67‐257)	22/22 (100%)	22/22 (100%)
Creatinine (n = 22)	9.0 (6.0‐15.9)	22/22 (100%)	22/22 (100%)
Phosphorus (n = 21)	13.3 (5.8‐22.6)	20/21 (95%)	20/21 (95%)
Albumin (n = 20)	2.6 (2.2‐3.3)	12/20 (60%)	12/20 (60%)
Sodium (n = 22)	143 (134‐149)	3/22 (14%)	3/22 (14%)
Potassium (n = 22)	5.2 (4.1‐8.1)	10/22 (45%)	10/22 (45%)
Total bilirubin (n = 22)	2.5 (0.1‐23.9)	14/22 (64%)	14/22 (64%)
Glucose (n = 21)	94 (55‐129)	1/21 (5%)	1/21 (5%)
Calcium (n = 21)	10.4 (8.8‐16.6)	7/21 (33%)	7/21 (33%)
ALP (n = 22)	428 (26‐939)	18/22 (82%)	18/22 (82%)
ALT (n = 22)	77 (2‐2705)	10/22 (45%)	10/22 (45%)
AST (n = 20)	82 (18‐1039)	12/20 (60%)	12/20 (60%)
GGT (n = 21)	6 (1‐18)	6/21 (29%)	6/21 (29%)
Cholesterol (n = 19)	204 (87‐375)	1/19 (5%)	1/19 (5%)

^a^ Absolute Count.

Abbreviations: ALP, alkaline phosphatase; ALT, alanine transaminase; AST, aspartate transaminase; GGT, gamma‐glutamyl transferase.

Eighteen dogs (82%) had elevations in all or 1 of the following: alkaline phosphatase (ALP), alanine transaminase (ALT), aspartate transaminase (AST), gamma‐glutamyl transferase (GGT) and bilirubin (see Table 1). On intake, 14 dogs (64%) were hyperbilirubinemic with a median serum bilirubin of 2.5 mg/dL (range, 0.1‐23.9 mg/dL). In most dogs (77%), bilirubin continued to rise with a median maxBili concentration of 8.0 mg/dL (range, 0.1‐41.6 mg/dL) occurring at a median of 3 days (range, 1‐10) days after admission.

Urinalysis abnormalities included proteinuria (20/22, 91%), glucosuria (16/22, 73%), pyuria (8/22, 36%) and bacteriuria (4/21, 19%). Four dogs had a UPC performed and had a median UPC of 1.7 (range, 1.2‐3.4). Six dogs (27%) had a positive urine culture on admission (2) or during hospitalization (4). Of the dogs that cultured positive during hospitalization, 3 had a previously negative urine culture. Cultured bacteria included *Escherichia coli* (3), *beta‐hemolytic streptococcus* (1), *Proteus miriabilis* (1) and *Enterococcus faecium* (1). One dog cultured *Klebsiella pneumoniae* (1) on both blood and urine cultures. Dogs with a new, positive urine culture during hospitalization and an active sediment were prescribed an antibiotic based on susceptibility testing, if they were not susceptible to an antibiotic in their current regimen.

All dogs had all CBCs performed during their hospitalization evaluated to assess for the minPLT. The median minPLT was 97 k/μL (range, 22‐264) and 6 dogs had a PLT <60 k/μL. Five dogs had PT and aPTT testing, of those 2 (40%) had a prolonged PT (median 8.9 seconds; range, 7.9‐19.0, reference range [RR]: 6.2‐9.3) and 4 (80%) of dogs had a prolonged aPTT (median 19.1 seconds; range, 15.8‐29.0; RR: 8.9‐16.3 seconds). Four dogs had a TEG performed and 3 were considered hypercoagulable and 1 was hypocoagulable based on interpretation of the Reaction Time (median 2.1 minutes; range, 0‐1.4; RR: 2.0‐7.0), *K* value (median 1.55 minutes; range, 0.8‐5.8, RR: 1.0‐4.0), α‐angle (median 68.6°; range, 48.0°‐77.0°; RR: 48.0‐77.0), Maximum Amplitude (median 64.2 mm; range, 30.8‐64.9; RR: 45.0‐64.0) and G (median 9.0K d/sc; range, 2.2K‐9.2K; RR: 3.9K‐8.4K).

Two dogs had echocardiograms. One dog was diagnosed with a significant, malignant ventricular arrhythmia secondary to hypokalemia (2.3 mEq/L; RR: 3.4‐4.9) secondary to severe diuresis during the recovery stage. One dog was diagnosed with left ventricular hypertrophy and a suspected thrombus in the right atrium.

### 3.5 Extracorporeal renal replacement therapy

Criteria to initiate extracorporeal therapies was an AKI and 1 or a combination of: inadequate urine production (16/22, 73%), volume overload (13/22, 59%) and hyperkalemia (10/22, 45%).

Sixty‐eight RRT sessions were performed. Session modalities included IHD (66) and CRRT (2). All dogs received at least 1 diffusive session with IHD, and 2 dogs received additional sessions with a CRRT platform. One dog received concurrent treatment with the VetResQ device. Sixty‐two RRT sessions (62/68, 91%) were performed with UFH and 6 (6/68, 9%) were performed with RCA. Ultrafiltration was included in 32 RRT sessions (32/68, 47%). Mannitol was preventively used in 15 sessions (22%) when there was a high risk of dialysis disequilibrium. The number of RRT sessions per dog were 1 (5, 23%), 2 (4, 18%), 3 (4, 18%), 4 (6, 27%), 5 (1, 5%), 6 (1, 5%) and 8 (1, 5%) with a median of 3 (range, 1‐8) sessions per dog.

Twenty‐two sessions (22/68, 32%) in 12 dogs had complications not requiring interventions. The most common complications were combinations of nonlife‐threatening arrhythmias including occasional VPCs (10), mild bradycardia (3), accelerated idioventricular rhythm (2) and sinus tachycardia (2). Other complications included nausea (4), epistaxis (1) and regurgitation (1). Twelve sessions (12/68, 18%) in 8 dogs had complications necessitating emergent interventions including lidocaine for treatment of ventricular tachycardia (2), mannitol administration due to suspected development of dialysis disequilibrium (1) and administration of fresh frozen plasma due to patient decline and uncontrollable bleeding (1). Ten sessions (10/68, 15%) in 7 dogs were emergently terminated due to severe arrhythmias (runs of VPCs [10], ventricular tachycardia [2], severe bradycardia [3]), concerns for dialysis disequilibrium (2) and agonal breathing (1). No dogs died during the sessions. Circuit complications causing early session termination included circuit clotting (3) and filter leakage (1).

### 3.6 Body systems affected

The number of body systems affected per dog were 1 (3/22, 14%), 2 (8/22, 36%), 3 (6/22, 27%) and 4 (5/22, 23%).

All dogs had kidney involvement based on azotemia and the severity of kidney dysfunction necessitating RRT. Twelve dogs (55%) had abnormalities on kidney imaging, including increased cortical echogenicity (9), reduced corticomedullary distinction (9), renomegaly (6), renal pelvic dilation (pelvic height >3 mm) (6), distortion of internal architecture (3) and irregular kidney surface (1).

All dogs were assessed for liver involvement based on elevations in 2 or more serum liver enzyme activities and bilirubin. Sixteen dogs (73%) had hepatic involvement. Of those, 9 dogs had abnormalities on hepatic imaging which included a hypoechoic liver (9) and hepatomegaly (5). Three dogs also had a hypoechoic pancreas and 7 had gallbladder sludge on abdominal ultrasound.

All dogs were assessed for pulmonary involvement based on persistent dyspnea and tachypnea. Twenty dogs had radiographs performed. Nine dogs had clinical signs of respiratory involvement and radiographic changes. One dog had severe dyspnea and tachypnea without radiographic changes. On necropsy, the dog had lung changes with diffuse, neutrophilic interstitial pneumonia with perivascular edema. These 10 dogs (45%) were classified as having pulmonary involvement. Of the 20 radiographed dogs, 10 (50%) had radiographic abnormalities including an alveolar pattern (6) with or without an interstitial pattern (10). No other dogs had clinical evidence of dyspnea and tachypnea without radiographic changes.

All dogs were assessed for coagulation involvement by documenting clinical evidence of an hemostatic disorder. Three dogs had evidence of bleeding including melena (1), epistaxis (1) and petechiation (1). One dog had evidence of clot formation after a clot was formed on the tip of the dialysis catheter. Six dogs had a PLT <60 k/μL (median 35 k/μL; range, 22‐49). Nine dogs had additional coagulation testing performed including PT and aPTT testing (5) and TEG (4). Of those, 8 were considered coagulopathic. Based on a combination of the testing above, 12 dogs (54%) had coagulation system involvement.

### 3.7 Factors associated with survival to discharge

Sixteen dogs (73%) survived to discharge. The median sCr at discharge for survivors was 2.2 mg/dL (range, 0.5‐6.3 mg/dL). Twelve dogs (75%) were azotemic at discharge.

Causes of death in non‐surviving dogs were cardiopulmonary arrest (CPA) (3/6) and euthanasia (3/6). All dogs with CPA arrested overnight and the underlying cause of CPA was undetermined. Reasons for euthanasia included persistent anuria in combination with respiratory distress (1), progressive hyperbilirubinemia and concern for disseminated intravascular coagulation (DIC) (1), and persistent anuria unresponsive to treatment (1). Necropsy on 1 dog revealed enterocolitis, necrotizing vasculitis with perivascular hemorrhage, interstitial edema and multifocal renal tubular degeneration and necrosis with proteinaceous casts, marked centrilobular congestion (nutmeg liver) with intracanalicular cholestasis, and diffuse, neutrophilic interstitial pneumonia with icterus and petechiation. Before death, 3 dogs had a normalized UOP and the median sCr was 7.2 mg/dL (range, 4.2‐13.5 mg/dL).

**FIGURE 1:**
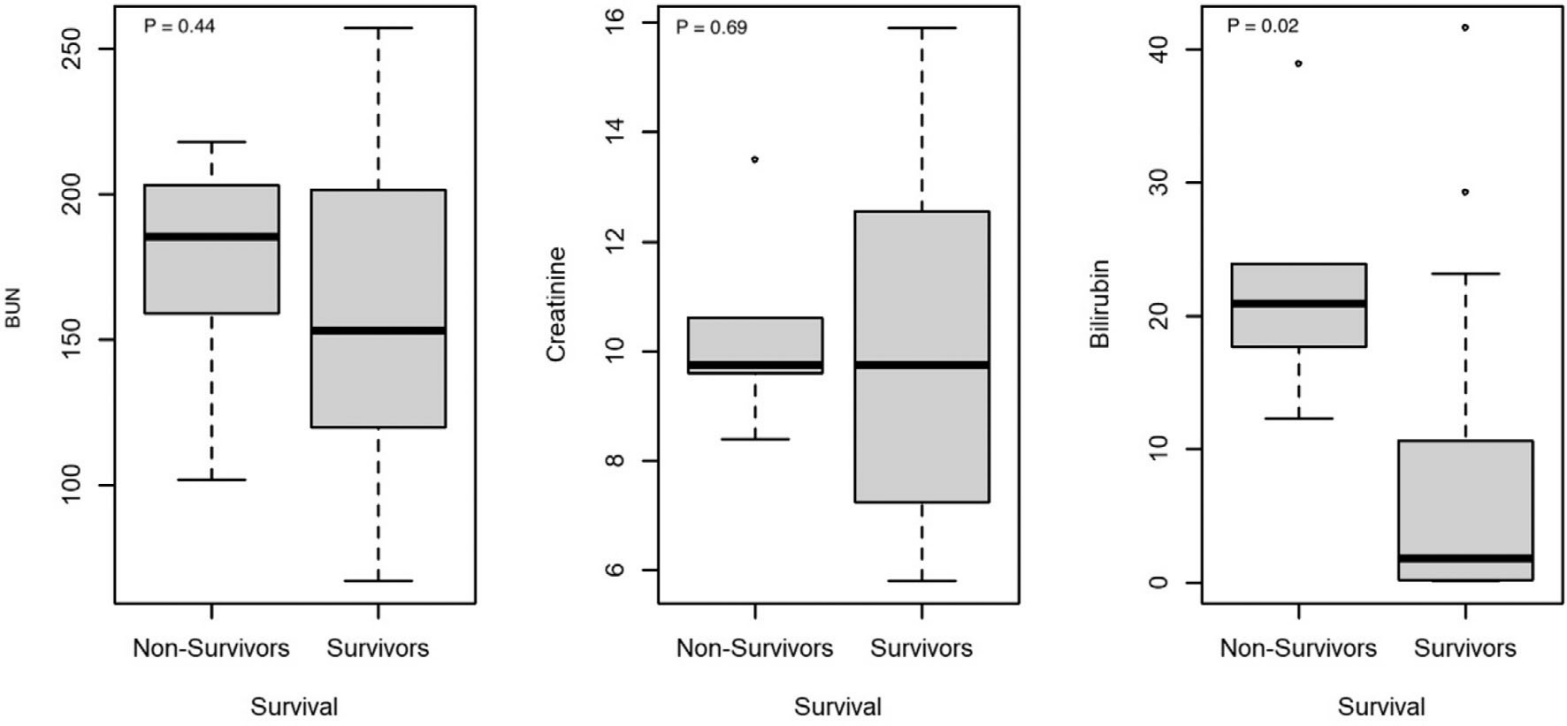
Comparison between maxBUN, maxCr and maxBili and between survivors and non‐survivors to discharge. The box represents the second and third quartiles. The horizontal line within the box represents the median. The whiskers represent the range and the circles indicate outliers.

**FIGURE 2:**
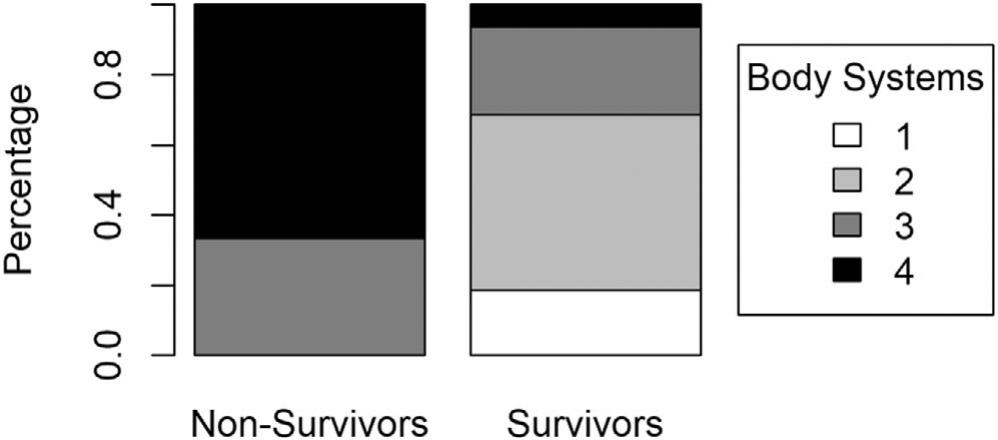
Comparison in number of body systems affected between survivors and non‐survivors to discharge.

Median maxBUN (*P* = .44) was not significantly different between survivors (153.0 mg/dL; range, 67‐257) and non‐survivors (185.5 mg/dL; range, 102‐218). Likewise, median maxCr (*P* = .69) was not significantly different between survivors (9.8 mg/dL; range, 6.2‐15.9) and non‐survivors (9.8 mg/dL; range, 8.4‐13.5). There was a significant difference in median maxBili (*P* = .02) between survivors (1.9 mg/dL; range, 0.1‐41.6) and non‐survivors (21.0 mg/dL; range, 12.3‐38.9) (Table 2, Figure 1). There was a significant difference between survival and the number of body systems affected (*P* = .01). Those who did not survive had more body systems affected (3 body systems affected 33.3%, 4 body systems affected 66.7%) compared with those who survived (1 body system affected 18.8%, 2 body systems affected 50%, 3 body systems affected 25%, 4 body systems affected 6.2%) (Figure 2, Table 2).

**TABLE 2:** Median (range) and significance of factors associated with survival to discharge.

Variables	Survivors (n = 16)	Non‐survivors (n = 6)	*P* value
Maximum BUN	153.0 mg/dL (67‐257)	185.5 mg/dL (102‐218)	.44
Maximum creatinine	9.8 mg/dL (6.2‐15.9)	9.8 mg/dL (8.4‐13.5)	.69
Maximum bilirubin	1.9 mg/dL (0.1‐41.6)	21.0 mg/dL (12.3‐38.9)	.02
No. body systems affected			.01
1	3 (18.8%)	0 (0%)	
2	8 (50.0%)	0 (0%)	
3	4 (25.0%)	2 (33.3%)	
4	1 (6.2%)	4 (66.7%)	

Spearman correlation analyses were used to assess the association between bilirubin and BUN and creatinine. There was no significant correlation between bilirubin and BUN (−0.08; *P* = .71) and creatinine (0.19, *P* = .40). Measures of effect could not be computed as continuous variables are presented as medians and due to the small sample size for body systems affected. Multivariate analysis (eg, logistic regression) was not possible due to small sample size and distribution of body systems affected.

### 3.8 Short term (≤30 days from discharge) outcome

From the 16 discharged dogs, 3 dogs (19%) were died ≤30 days from discharge. Causes of death included: declining patient condition in an RRT‐dependent dog (1), sudden death (1) and progressive azotemia (1). None of these dogs had necropsies performed.

### 3.9 Long term outcome (≥6 months)

Of the 13 dogs that survived >30 days from discharge, 12 were alive ≥6 months from discharge and 8 were alive at the time of writing:

The 8 dogs alive at time of writing were staged according to IRIS CKD staging guidelines^20^ as Stage I (5) and Stage II (3). The median sCr was 1.3 mg/dL (range, 0.6‐2.2 mg/dL). Creatinine normalization occurred in 4 out of the 8 dogs (50%). Staging occurred a median of 13 months (range, 6‐24 months) after discharge.

The 4 dogs not alive at time of writing were euthanized due to suspected kidney disease at 3 years (2), 2 years (1) and 7 months (1) after discharge. Serum creatinine before death was not available. The dog euthanized 7 months after discharge was newly diagnosed with metastatic mast cell neoplasia. Before euthanasia, the dog's serum creatinine was 1.0 mg/dL. None of these dogs had necropsies performed.

## 4 DISCUSSION

Our study describes the outcomes of a cohort of dogs with severe clinical manifestations of leptospirosis that had access to RRT. Approximately ¾ of the dogs survived to discharge and of those, approximately ¾ were alive at 6 months from discharge. Despite severe azotemia on presentation, there was no significant difference in maxBUN or maxCr between survivors and non‐survivors to discharge. Non‐survivors to discharge had a significantly higher number of body systems affected in comparison to survivors to discharge. Although 75% of survivors were azotemic at discharge, kidney values continued to improve over time and normalized in 50% of long‐term surviving cases.

Our study's survival rate to discharge was 73%. Other studies have found survival rates between dogs with AKI‐L who did not require RRT (68%)^6^, dogs with AKI of multiple etiologies treated with RRT (60%)^23^, and dogs with AKI‐L that received RRT (86%).^5^ The difference in survival rates might be attributed to differences in dog cohorts, fluid status and serovars. Most dogs in our study had negative prognostic factors associated with survival to discharge including overhydration (59%) and anuria or oliguria (64%).^5^, ^6^, ^22^, ^23^ Our dog cohort had MAT titers highest to Bratislava (41%) and Gryppotyphosa (41%) serovars which differs from other studies.^5^ We suspect that the difference in predominating titers might be geographical and previous studies have suggested only a minor clinically relevant differences between serovars and the poor ability of antibody tests to predict infecting serovar.^5^, ^24^, ^25^, ^26^ The good survival rate in our AKI‐L dogs with severe clinical presentation highlights RRT success when available.

Maximum blood urea nitrogen and maxCr were not associated with survival to discharge. Studies have shown that azotemia severity might be associated with short‐term survival in dogs with leptospirosis, dogs with AKI and in dogs with AKI of varying etiologies requiring RRT.^6^, ^22^, ^23^ Although azotemia severity might have been underestimated due to the suppressive effect of hyperbilirubinemia on sCr, our study did not reveal an association between bilirubin and BUN or creatinine. According to our chemistry analyzer's manufacturer, creatinine suppression occurs with bilirubin levels >20 mg/dL, as seen in 6 dogs (see Data S1). Similarly to our study, other studies in RRT dogs revealed that azotemia severity is not a negative prognostic factor.^5^, ^11^ The lack of association between maxBUN, maxCr and survival suggests that the severity of azotemia does not reflect the reversibility of kidney dysfunction.^22^, ^23^ Leptospirosis, a potentially reversible cause of AKI, carries a more favorable prognosis in contrast to other causes of irreversible AKI where a greater degree of azotemia is associated with a worse prognosis.^22^, ^23^, ^27^ Therefore, prognosis for dogs with AKI‐L that can have access to RRT should not be determined based on serum BUN and creatinine.

Non‐survivors to discharge had a significantly higher number of body systems affected compared with survivors. Other studies have identified that multisystemic involvement is associated with a worse outcome.^6^, ^23^ Leptospirosis has a strong ability to induce proinflammatory cytokines resulting in multisystemic disease.^28^ Multisystemic dysfunction adds to the complexity of case management due to the challenges associated with balancing acute liver injury, coagulation status, respiratory status and other life‐threatening systemic complications in conjunction with the AKI. These challenges are illustrated by the fact that multisystemic disease was cited as a contributing factor in the decision for euthanasia in the dogs that did not survive to discharge. In human medicine, cytokine filters have been used to reduce inflammatory mediators in patients undergoing RRT with inflammatory conditions.^29^, ^30^ Based on the aforementioned principle, 1 dog received treatment with a newly available cytokine filter. The effect of the cytokine filter on the dog's outcome is unclear. There are ongoing studies evaluating the utility of blood purification therapy in AKI‐L dogs at our institution.

Bilirubin was higher in non‐survivors compared with survivors. Severe hyperbilirubinemia might reflect progressive disease, as RRT does not remove unconjugated bilirubin, which is plasma protein bound.^31^ The suspected mechanism of hyperbilirubinemia in leptospirosis is the invasion of hepatocytes' intracellular junctions by leptospires, causing bile leakage.^32^ However, our dog population might have had other concomitant causes of hyperbilirubinemia including hemolysis, pancreatitis, gallbladder disease, cytokine storm and its' sequela, DIC.^28^, ^33^ Hyperbilirubinemia is associated with mortality in dogs and humans as it reflects manifestations of severe multisystemic disease.^6^, ^22^, ^31^ In humans, hyperbilirubinemia might also worsen kidney injury through bilirubin cast deposition.^34^ Our study's results are in accordance with previous studies as hyperbilirubinemia was significantly associated with survival. However, as multivariate analysis was unable to be performed, the possibility that the number of body systems affected and hyperbilirubinemia might be confounding in our study cannot be excluded.

Dogs with AKI‐L receiving RRT have an overall good survival rate. Despite 75% of dogs remaining azotemic at discharge, 50% of dogs alive at long‐term follow‐up had a normalization in sCr. A study on dogs managed conservatively for leptospirosis documented normalization in sCr either at discharge or at follow‐up in 75% of dogs.^6^ Another study looking at dogs with AKI of varying etiologies found Scr normalization in 55% of dogs at discharge, and an additional 20% at follow‐up.^35^ Although dogs remaining azotemic at and following discharge are higher than previously described, no studies have looked uniquely at normalization of kidney values in AKI‐L dogs requiring RRT. The dogs in our study were likely to have sustained worse kidney injury as they met indications for RRT while the others were treated conservatively. Additionally, long‐term follow‐up in our study was variable and other factors including undocumented additional AKIs and concurrent diseases might have exacerbated underlying kidney dysfunction. Kidney recovery and repair might take weeks to months and continued improvement and further normalization is possible.^22^, ^35^

Limitations of this study included its retrospective design, small cohort of dogs and lack of standardization in diagnostics performed and long‐term follow‐up. Some diagnostics were performed within 24 hours of but not before RRT initiation due to emergent RRT sessions that occurred overnight. The number of body systems affected might have been underestimated as dogs were not uniformly assessed for the body systems affected. Dogs might have also had additional systems affected (ie, myocarditis, vasculitis, enteritis) although this was not included due to the difficulty in distinguishing clinical signs from uremic signs and severe electrolyte disturbances and the lack of standardized diagnostics. Additionally, hyperbilirubinemia and number of body systems affected might be correlated or confounding but multivariable analysis could not be performed. Six dogs in the study had bilirubin levels >20 mg/dL, sCr might have been suppressed in these dogs. It is unclear the degree in which concurrent diseases, age and owner finances might have negatively affected survival to discharge. Due to a small study cohort, multivariate analysis was unable to be performed to assess for the effect of other variables including patient age, underlying CKD, fluid‐status and co‐morbidities on survival to discharge. There was no standardized follow‐up protocol for the dogs, therefore other factors including prior kidney disease, additional AKIs, medications and additional co‐morbidities might have played a role in long‐term patient outcome. Surviving dogs were only staged using sCr and additional biomarkers or repeat imaging were not used. Due to 4‐year study duration, dogs might have been in various stages of kidney recovery when they were staged. Finally, our study describes the outcome of only a small cohort of dogs that had access to RRT. Outcomes might be different for other causes of AKI, dogs with AKI‐L but not requiring RRT or dogs with AKI‐L who require RRT but do not have access to RRT.

In conclusion, our study presents the short and long‐term outcomes of a specific cohort of 22 dogs with AKI‐L requiring RRT. Survival to discharge was 73% and 75% of survivors were alive at long‐term follow‐up (≥6 months). Seventy‐five percent of dogs were azotemic at discharge and 50% of long‐term survivors were azotemic on follow‐up, thus long‐term CKD is common in these dogs. Maximum blood urea nitrogen and maxCr were not associated with survival to discharge and should not be used to prognosticate in this group of dogs. Multiorgan involvement and hyperbilirubinemia might have negatively affected survival to discharge as these complications might not be correctable with RRT.

## CONFLICT OF INTEREST DECLARATION

Mary Anna Labato consults for Boehringer Ingelheim. No other authors declare a conflict of interest.

## OFF‐LABEL ANTIMICROBIAL DECLARATION

Authors declare no off‐label use of antimicrobials.

## INSTITUTIONAL ANIMAL CARE AND USE COMMITTEE (IACUC) OR OTHER APPROVAL DECLARATION

Authors declare no IACUC or other approval was needed.

## HUMAN ETHICS APPROVAL DECLARATION

Authors declare human ethics approval was not needed for this study.

## Supporting information


**Data S1.** Supporting Information.
