# Erratum for “Retrospective evaluation of 22 dogs with leptospirosis treated with extracorporeal renal replacement therapies (2018‐2021)”

**DOI:** 10.1111/jvim.17146

**Published:** 2024-07-17

First published: 15 April 2020, https://doi.org/10.1111/jvim.16998

Volume 38, Issue 2

Section 6 Discussion, 2nd paragraph, 2nd sentence, the 2nd reference is 22 and not 23. The sentence should be as follows. “Other studies have found survival rates between dogs with AKI‐L who did not require RRT (68%),^6^ dogs with AKI of multiple etiologies treated with RRT (60%),^22^ and dogs with AKI‐L that received RRT (86%).^5^”

In Table 1, the Reference Range column values are not correct. The correct Table 1 is this.

**TABLE 1:** Clinical‐pathological results of dogs with leptospirosis within 24 hours of first treatment with RRT.

Variable	Median (range)	Proportion (%) abnormal values	Reference range
White blood cell count (n = 22)	17.5 (10.5‐34.6)	16/22 (78%)	4.40‐15.10 K/μL
Hematocrit (n = 22)	37.0 (23.0‐54.9)	16/22 (78%)	39‐55%
Platelet (n = 20)	115 (46‐263)	15/20 (75%)	173‐486 K/μL
Reticulocyte^a^ (n = 22)	27.8 (6.7‐78.4)	—	14.7‐113.7 K/μL
Neutrophil^a^ (n = 20)	14.505 (8.000‐27.656)	15/20 (75%)	2.800‐11.500 K/μL
Lymphocyte^a^ (n = 22)	1.46 (0.38‐7.65)	7/22 (32%)	1.00‐4.80 K/μL
Monocyte^a^ (n = 22)	1.10 (0.30‐2.62)	7/22 (32%)	0.10‐1.50 K/μL
BUN (n = 22)	152 (67‐257)	22/22 (100%)	8‐30 mg/dL
Creatinine (n = 22)	9.0 (6.0‐15.9)	22/22 (100%)	0.6‐2.0 mg/dL
Phosphorus (n = 21)	13.3 (5.8‐22.6)	20/21 (95%)	2.6‐7.2 mg/dL
Albumin (n = 20)	2.6 (2.2‐3.3)	12/20 (60%)	2.8‐4.0 g/dL
Sodium (n = 22)	143 (134‐149)	3/22 (14%)	140‐150 mEq/L
Potassium (n = 22)	5.2 (4.1‐8.1)	10/22 (45%)	3.7‐5.4 mEq/L
Total bilirubin (n = 22)	2.5 (0.1‐23.9)	14/22 (64%)	0.10‐0.3 mg/dL
Glucose (n = 21)	94 (55‐129)	1/21 (5%)	67‐135 mg/dL
Calcium (n = 21)	10.4 (8.8‐16.6)	7/21 (33%)	9.4‐11.3 mg/dL
ALP (n = 22)	428 (26‐939)	18/22 (82%)	12‐127 U/L
ALT (n = 22)	77 (2‐2705)	10/22 (45%)	14‐86 U/L
AST (n = 20)	82 (18‐1039)	12/20 (60%)	9‐54 U/L
GGT (n = 21)	6 (1‐18)	6/21 (29%)	0‐10 U/L
Cholesterol (n = 19)	204 (87‐375)	1/19 (5%)	82‐355 mg/dL

Abbreviations: ALP, alkaline phosphatase; ALT, alanine transaminase; AST, aspartate transaminase; GGT, gamma‐glutamyl transferase.

^a^ Absolute Count.

