# Supplementary material for: Retrospective evaluation of 22 dogs with leptospirosis treated with extracorporeal renal replacement therapies (2018‐2021)
Source: J Vet Intern Med. 2024 Feb 9;38(2):1051–9. doi: 10.1111/jvim.16998 (PMC10937474; doi:10.1111/jvim.16998)
Supplement: Supplementary file 1 — Data S1. Supporting Information. [file JVIM-38-1051-s001.pdf]

CREP2

cobas®

Creatinine plus ver.2

Substrates

Order information

REF	CONTENT		Analyzer(s) on which **cobas c** pack(s) can be used
**03263991** 190	Creatinine plus ver.2 (250 tests)	System-ID 07 6612 7	COBAS INTEGRA 400 plus

Materials required (but not provided):

**10759350** 190	Calibrator f.a.s. (12 × 3 mL)	System-ID 07 3718 6	
**10759350** 360	Calibrator f.a.s. (12 × 3 mL, for USA)	System-ID 07 3718 6	
**12149435** 122	Precinorm U plus (10 × 3 mL)	System-ID 07 7999 7	
**12149435** 160	Precinorm U plus (10 × 3 mL, for USA)	System-ID 07 7999 7	
**12149443** 122	Precipath U plus (10 × 3 mL)	System-ID 07 8000 6	
**12149443** 160	Precipath U plus (10 × 3 mL, for USA)	System-ID 07 8000 6	
**05117003** 190	PreciControl ClinChem Multi 1 (20 × 5 mL)	System-ID 07 7469 3	
**05947626** 190	PreciControl ClinChem Multi 1 (4 × 5 mL)	System-ID 07 7469 3	
**05947626** 160	PreciControl ClinChem Multi 1 (4 × 5 mL, for USA)	System-ID 07 7469 3	
**05117216** 190	PreciControl ClinChem Multi 2 (20 × 5 mL)	System-ID 07 7470 7	
**05947774** 190	PreciControl ClinChem Multi 2 (4 × 5 mL)	System-ID 07 7470 7	
**05947774** 160	PreciControl ClinChem Multi 2 (4 × 5 mL, for USA)	System-ID 07 7470 7	
**03121313** 122	Precinorm PUC (4 × 3 mL)	System-ID 07 6756 5	
**03121291** 122	Precipath PUC (4 × 3 mL)	System-ID 07 6757 3	

English

System information

Test CRE2, test ID 0-612 (serum, plasma)

Test CRE2U, test ID 0-512 (urine)

Intended use

In vitro test for the quantitative determination of the creatinine concentration in human serum, plasma, and urine on COBAS INTEGRA systems.

Summary[1,2,3,4,5]

Chronic kidney disease is a worldwide problem that carries a substantial risk for cardiovascular morbidity and death. Current guidelines define chronic kidney disease as kidney damage or glomerular filtration rate (GFR) less than 60 mL/min per 1.73 m^2 for three months or more, regardless of cause.

The assay of creatinine in serum or plasma is the most commonly used test to assess renal function. Creatinine is a break-down product of creatine phosphate in muscle, and is usually produced at a fairly constant rate by the body (depending on muscle mass). It is freely filtered by the glomeruli and, under normal conditions, is not re-absorbed by the tubules to any appreciable extent. A small but significant amount is also actively secreted.

Since a rise in blood creatinine is observed only with marked damage of the nephrons, it is not suited to detect early stage kidney disease. A considerably more sensitive test and better estimation of glomerular filtration rate (GFR) is given by the creatinine clearance test based on creatinine's concentration in urine and serum or plasma, and urine flow rate. For this test a precisely timed urine collection (usually 24 hours) and a blood sample are needed. However, since this test is prone to error due to the inconvenient collection of timed urine, mathematical attempts to estimate GFR based only on the creatinine concentration in serum or plasma have been made. Among the various approaches suggested, two have found wide recognition: that of Cockroft and Gault and that based on the results of the MDRD trial. While the first equation was derived from data obtained with the conventional Jaffé method, a newer version of the second is usable for IDMS-traceable creatinine methods. Both are applicable for adults. In children, the Bedside Schwartz formula should be used. [6,7,8,9]

In addition to the diagnosis and treatment of renal disease, the monitoring of renal dialysis, creatinine measurements are used for the calculation of the fractional excretion of other urine analytes (e. g. albumin, α-amylase). Numerous methods were described for determining creatinine. Automated assays established in the routine laboratory include the Jaffé alkaline picrate method in various modifications, as well as enzymatic tests.

Test principle

Enzymatic colorimetric method

This enzymatic method is based on the conversion of creatinine with the aid of creatininase, creatinase, and sarcosine oxidase to glycine, formaldehyde and hydrogen peroxide. Catalyzed by peroxidase the liberated hydrogen peroxide reacts with 4-aminophenazone and HTIB[a] to form a quinone imine chromogen. The color intensity of the quinone imine chromogen formed is directly proportional to the creatinine concentration in the reaction mixture.

$$\text{creatinine} + H_2O \xrightarrow{\text{creatininase}} \text{creatine}$$

$$\text{creatine} + H_2O \xrightarrow{\text{creatinase}} \text{sarcosine} + \text{urea}$$

$$\text{sarcosine} + O_2 + H_2O \xrightarrow{\text{SOD}} \text{glycine} + \text{HCHO} + H_2O_2$$

$$H_2O_2 + \text{4-aminophenazone} + \text{HTIB} \xrightarrow{\text{POD}} \text{quinone imine chromogen} + H_2O + \text{HI}$$

Creatine of the sample is destroyed by creatinase, SOD and catalase during incubation in R1.

a) 2,4,6-triiodo-3-hydroxybenzoic acid

Reagents - working solutions

R1 TAPS[b] buffer: 30 mmol/L, pH 8.1; creatinase (microorganisms): ≥ 332 µkat/L; sarcosine oxidase (microorganisms): ≥ 132 µkat/L; ascorbate oxidase (microorganisms): ≥ 33 µkat/L; catalase (microorganisms): ≥ 1.67 µkat/L; HTIB: 1.2 g/L; detergents; preservative

SR TAPS[b] buffer: 50 mmol/L, pH 8.0; creatininase (microorganisms): ≥ 498 µkat/L; peroxidase (horseradish): ≥ 16.6 µkat/L; 4-aminophenazone: 0.5 g/L; potassium hexacyanoferrate (II): 60 mg/L; detergent; preservative

b) N-Tris(hydroxymethyl)methyl-3-aminopropanesulfonic acid

R1 is in position B and SR is in position C.

Precautions and warnings

For in vitro diagnostic use for health care professionals. Exercise the normal precautions required for handling all laboratory reagents.

Infectious or microbial waste:
Warning: handle waste as potentially biohazardous material. Dispose of waste according to accepted laboratory instructions and procedures.

Environmental hazards:
Apply all relevant local disposal regulations to determine the safe disposal.

Safety data sheet available for professional user on request.

For USA: Caution: Federal law restricts this device to sale by or on the order of a physician.

Reagent handling

Ready for use

Storage and stability

Shelf life at 2-8 °C	See expiration date on **cobas c** pack label
On-board in use at 10-15 °C	8 weeks

Specimen collection and preparation

For specimen collection and preparation only use suitable tubes or collection containers.

Only the specimens listed below were tested and found acceptable.
Serum
Plasma: Li-heparin, Na-heparin, K_3-EDTA, or Na_2-EDTA plasma

The sample types listed were tested with a selection of sample collection tubes that were commercially available at the time of testing, i.e. not all available tubes of all manufacturers were tested. Sample collection systems from various manufacturers may contain differing materials which could affect the test results in some cases. When processing samples in primary tubes (sample collection systems), follow the instructions of the tube manufacturer.

Urine: Collect urine without using additives. If urine must be collected with a preservative for other analytes, only hydrochloric acid (14-47 mmol/L urine, e.g. 5 mL 10 % HCl or 5 mL 30 % HCl per liter urine) or boric acid (81 mmol/L, e.g. 5 g per liter urine) may be used.
Urine samples are automatically prediluted 1:20 (1+19) with water by the instrument.

Centrifuge samples containing precipitates before performing the assay.

See the limitations and interferences section for details about possible sample interferences.

Sample stability claims were established by experimental data by the manufacturer or based on reference literature and only for the temperatures/time frames as stated in the method sheet. It is the responsibility of the individual laboratory to use all available references and/or its own studies to determine specific stability criteria for its laboratory.

Stability in *serum/plasma*:[10]	7 days at 15-25 °C
	7 days at 2-8 °C
	3 months at (-15)-(-25) °C
Stability in *urine* (without preservative):[10]	2 days at 15-25 °C
	6 days at 2-8 °C
	6 months at (-15)-(-25) °C
Stability in *urine* (with preservative):	3 days at 15-25 °C
	8 days at 2-8 °C
	3 weeks at (-15)-(-25) °C

Materials provided

See “Reagents – working solutions” section for reagents.

Assay

For optimum performance of the assay follow the directions given in this document for the analyzer concerned. Refer to the appropriate operator’s manual for analyzer-specific assay instructions.

Applications for serum, plasma, and urine

Test definition

Measuring mode	Absorbance
Abs. calculation mode	Endpoint
Reaction direction	Increase
Wavelength A/B	552/659 nm
Calc. first/last	35/65
Serum, plasma	
Reaction mode	R1-S-SR
Unit	µmol/L
Urine	
Reaction mode	D-R1-S-SR
Predilution factor	20
Unit	mmol/L

Pipetting parameters

		Diluent (H_2O)
Serum, plasma		Diluent (H_2O)
R1	77 µL	
Sample	2 µL	5 µL
SR	38 µL	
Total volume	122 µL	
Urine		Diluent (H_2O)
R1	77 µL	
Sample	2 µL	5 µL
SR	38 µL	
Total volume	122 µL	

Calibration

Calibrator	Calibrator f.a.s.
	Use deionized water as zero calibrator.
Calibration mode	Linear regression
Calibration replicate	Duplicate recommended
Calibration interval	Each lot and as required following quality control procedures

Calibration interval may be extended based on acceptable verification of calibration by the laboratory.

Traceability: This method has been standardized against ID/MS.

Quality control

Quality control serum/plasma	Precinorm U plus or PreciControl ClinChem Multi 1
	Precipath U plus or PreciControl ClinChem Multi 2
Quality control urine	Precinorm PUC or Precipath PUC
Control interval	24 hours recommended
Control sequence	User defined
Control after calibration	Recommended

For quality control, use control materials as listed in the “Order information” section. In addition, other suitable control material can be used.

The control intervals and limits should be adapted to each laboratory’s individual requirements. Values obtained should fall within the defined limits. Each laboratory should establish corrective measures to be taken if values fall outside the defined limits.

Follow the applicable government regulations and local guidelines for quality control.

Calculation

The COBAS INTEGRA 400 plus analyzer automatically calculates the analyte concentration of each sample. For more details, please refer to Data Analysis in the Online Help.

Conversion factor: µmol/L × 0.0113 = mg/dL

Limitations - interference

Criterion: Recovery within ± 10 % of initial value.

Serum, plasma

Icterus:[11] No significant interference up to an I index of 20 for conjugated and unconjugated bilirubin (approximate conjugated and unconjugated bilirubin concentration: 340 µmol/L or 20 mg/dL).

Hemolysis:[11] No significant interference up to an H index of 800 (approximate hemoglobin concentration: 497 µmol/L or 800 mg/dL).

Lipemia (Intralipid):[11] No significant interference up to an L index of 1000. There is poor correlation between the L index (corresponds to turbidity) and triglycerides concentration.

Drugs: No interference was found at therapeutic concentrations using common drug panels.[12,13]
Exceptions: Rifampicin, levodopa and calcium dobesilate cause artificially low creatinine levels at the tested drug level while N-ethylglycine and DL-proline at a concentration of > 1 mmol/L causes falsely high results.
2-Phenyl-1,3-indandion (Phenindion) at therapeutic concentrations interferes with the assay.
Dicynone (Ethamsylate) at therapeutic concentrations may lead to false-low results.[14]

As tested according to CLSI recommendation methyldopa causes artificially low creatinine results.[15]

Ascorbic acid: No significant interference up to an ascorbic acid concentration of 1.70 mmol/L (30 mg/dL)

Acetaminophen intoxications are frequently treated with N-Acetylcysteine. N-Acetylcysteine at a plasma concentration above 333 mg/L and the Acetaminophen metabolite N-acetyl-p-benzoquinone imine (NAPQI) independently may cause falsely low results.

Venipuncture should be performed prior to the administration of Metamizole. Venipuncture immediately after or during the administration of Metamizole may lead to falsely low results. A significant interference may occur at plasma Metamizole concentrations above 0.05 mg/mL.

In very rare cases, gammopathy, in particular type IgM (Waldenström's macroglobulinemia), may cause unreliable results.[16]

No significant interference up to a creatine concentration of 0.38 mmol/L (50 mg/L).

Urine

Drugs: No interference was found at therapeutic concentrations using common drug panels.[13]
Exceptions: Methyldopa and levodopa causes artificially low results.
Dicynone (Ethamsylate) at therapeutic concentrations may lead to false-low results.

Acetaminophen, Acetylcysteine and Metamizole are metabolized quickly. Therefore, interference from these substances is unlikely but cannot be excluded.

No significant interference up to a creatine concentration of 3.05 mmol/L (40 mg/dL).

Criterion: Recovery within ± 10 % of initial value at a creatinine concentration of 2500 µmol/L (28.3 mg/dL).

Urea: No significant interference from urea up to a concentration of 2100 mmol/L (12612 mg/dL).

High homogentisic acid concentrations in urine samples lead to false results.

Estimation of the Glomerular Filtration Rate (GFR) on the basis of the Schwartz Formula can lead to an overestimation.[17]

For diagnostic purposes, the results should always be assessed in conjunction with the patient's medical history, clinical examination and other findings.

ACTION REQUIRED
Special Wash Programming: The use of special wash steps is mandatory when certain test combinations are run together on COBAS INTEGRA analyzers. Refer to the CLEAN Method Sheet for further instructions and for the latest version of the Extra wash cycle list.
Where required, special wash/carry-over evasion programming must be implemented prior to reporting results with this test.

Limits and ranges

Measuring range

Serum/plasma

5-2700 µmol/L (0.057-30.5 mg/dL)

Determine samples having higher concentrations via the rerun function. Dilution of samples via the rerun function is a 1:10 dilution. Results from samples diluted using the rerun function are automatically multiplied by a factor of 10.

Urine

0.1-40 mmol/L (1.13-452 mg/dL)

Determine samples having higher concentrations via the rerun function. Dilution of samples via the rerun function is a 1:5 dilution. Results from samples diluted using the rerun function are automatically multiplied by a factor of 5.

Lower limits of measurement

Serum/plasma

Lower detection limit of the test:
5 µmol/L (0.057 mg/dL)

The lower detection limit represents the lowest measurable analyte level that can be distinguished from zero. It is calculated as the value lying 3 standard deviations above that of a zero sample (zero sample + 3 SD, repeatability, n = 30).

Urine

Lower detection limit of the test:
0.1 mmol/L (1.13 mg/dL)

The lower detection limit represents the lowest measurable analyte level that can be distinguished from zero. It is calculated as the value lying 3 standard deviations above that of a zero sample (zero sample + 3 SD, repeatability, n = 30).

Expected values

Serum, plasma

Adults[18]		
Females	45-84 µmol/L	(0.51-0.95 mg/dL)
Males	59-104 µmol/L	(0.67-1.17 mg/dL)
Children[19]		
Neonates (premature)	29-87 µmol/L	(0.33-0.98 mg/dL)
Neonates (full term)	27-77 µmol/L	(0.31-0.88 mg/dL)
2- < 12 m	14-34 µmol/L	(0.16-0.39 mg/dL)
1- < 3 y	15-31 µmol/L	(0.18-0.35 mg/dL)
3- < 5 y	23-37 µmol/L	(0.26-0.42 mg/dL)
5- < 7 y	25-42 µmol/L	(0.29-0.47 mg/dL)
7- < 9 y	30-47 µmol/L	(0.34-0.53 mg/dL)
9- < 11 y	29-56 µmol/L	(0.33-0.64 mg/dL)
11- < 13 y	39-60 µmol/L	(0.44-0.68 mg/dL)
13- < 15 y	40-68 µmol/L	(0.46-0.77 mg/dL)

Roche has not evaluated reference ranges in a pediatric population.

Urine

1st morning urine[18]		
Females	2.55-20.0 mmol/L	(29-226 mg/dL)
Males	3.54-24.6 mmol/L	(40-278 mg/dL)
24h urine[20]		
Females	6-13 mmol/24 h	(720-1510 mg/24 h)
Males	9-19 mmol/24 h	(980-2200 mg/24 h)
Creatinine clearance[20]	66-143 mL/min	

Each laboratory should investigate the transferability of the expected values to its own patient population and if necessary determine its own reference ranges.

Specific performance data

Representative performance data on the COBAS INTEGRA analyzers are given below. Results obtained in individual laboratories may differ.

Precision

Serum/plasma

Precision was determined using human samples and controls in an internal protocol with repeatability (n = 21) and intermediate precision (1 aliquot per run, 1 run per day, 21 days). The following results were obtained:

Repeatability	Level 1	Level 2
Mean	89.7 µmol/L (1.02 mg/dL)	329 µmol/L (3.72 mg/dL)
CV	1.6 %	0.7 %

Intermediate precision	Level 1	Level 2
Mean	92.0 µmol/L (1.04 mg/dL)	335 µmol/L (3.79 mg/dL)
CV	1.3 %	0.9 %

Urine

Precision was determined using human samples and controls in an internal protocol with repeatability (n = 21) and intermediate precision (1 aliquot per run, 1 run per day, 10 days). The following results were obtained:

Repeatability	Level 1	Level 2
Mean	9.35 mmol/L (106 mg/dL)	20.5 mmol/L (232 mg/dL)
CV	0.8 %	1.8 %

Intermediate precision	Level 1	Level 2
Mean	9.55 mmol/L (108 mg/dL)	21.1 mmol/L (238 mg/dL)
CV	2.0 %	3.9 %

Method comparison

Creatinine values for human serum, plasma and urine samples obtained on a COBAS INTEGRA 400 analyzer using the COBAS INTEGRA Creatinine plus ver.2 reagent (y) were compared with those determined using the commercially available Creatinine plus reagent on a Roche/Hitachi 917 analyzer (x).

Serum/plasma

**Roche/Hitachi 917 analyzer**	Sample size (n) = 53
Corr. coefficient (r)	0.999
Lin. regression	y = 1.010x + 1.13 µmol/L
Passing/Bablok[21]	y = 1.013x - 1.50 µmol/L

The sample concentrations were between 53 and 2300 µmol/L (0.60 and 26.1 mg/dL).

Urine

**Roche/Hitachi 917 analyzer**	Sample size (n) = 54
Corr. coefficient (r)	0.998
Lin. regression	y = 0.935x + 0.625 mmol/L
Passing/Bablok[21]	y = 0.960x + 0.308 mmol/L

The sample concentrations were between 1.3 and 36 mmol/L (14.7 and 406 mg/dL).

References

1 Thomas C, Thomas L. Labordiagnostik von Erkrankungen der Nieren und ableitenden Harnwege. In: Thomas L, ed. Labor und Diagnose, 6th ed. Frankfurt/Main: TH-Books 2005;520-585.

2 Lamb E, Newman DJ, Price CP. Kidney function tests In: Burtis CA, Ashwood ER, Bruns DE. Tietz textbook of clinical chemistry and molecular diagnostics. 4th ed. St.Louis, MO: Elsevier Saunders 2006;797-835.

3 http://www.kidney.org/

4 http://www.nkdep.nih.gov/.2004;69:19643-19673.

5 Lamb EJ, Tomson CRV, Roderick PJ. Estimating kidney function in adults using formulae. Ann Clin Biochem 2005;42:321-345.

6 Miller WG. Editorial on Estimating glomerular filtration rate. Clin Chem Lab Med 2009;47(9):1017-1019.

7 Schwartz GJ, Muñoz A, Schneider MF, et al. New Equations to Estimate GFR in Children with CKD. J Am Soc Nephrol 2009;20:629-637.

8 Schwartz GJ, Work DF. Measurement and Estimation of GFR in Children and Adolescents. Clin J Am Soc Nephrol 2009;4:1832–1843.

9 Staples A, LeBlond R, Watkins S, et al. Validation of the revised Schwartz estimating equation in a predominantly non-CKD population. Pediatr Nephrol 2010 Jul 22;25:2321-2326.

10 Guder WG, da Fonseca-Wollheim F, Heil W, et al. Die Qualität Diagnostischer Proben, 6. Aufl. Heidelberg: BD Diagnostics, 2009.

11 Glick MR, Ryder KW, Jackson SA. Graphical Comparisons of Interferences in Clinical Chemistry Instrumentation. Clin Chem 1986;32:470-475.

12 Breuer J. Report on the Symposium "Drug effects in Clinical Chemistry Methods". Eur J Clin Chem Clin Biochem 1996;34:385-386.

13 Sonntag O, Scholer A. Drug interference in clinical chemistry: recommendation of drugs and their concentrations to be used in drug interference studies. Ann Clin Biochem 2001;38:376-385.

14 Dastych M, Wiewiorka O, Benovska M. Ethamsylate (Dicynone) Interference in Determination of Serum Creatinine, Uric Acid, Triglycerides, and Cholesterol in Assays Involving the Trinder Reaction; In Vivo and In Vitro. Clin Lab 2014;60:1373-1376.

15 CLSI. Interference testing in Clinical Chemistry; Approved Guideline-Second Edition. CLSI document EP7-A2, Wayne, Pennsylvania, 2005.

16 Bakker AJ, Mücke M. Gammopathy interference in clinical chemistry assays: mechanisms, detection and prevention. Clin Chem Lab Med 2007;45(9):1240-1243.

17 Filler G, Priem F, Lepage N, et al. β-Trace Protein, Cystatin C, β2-Microglobulin, and Creatinine Compared for Detecting Impaired Glomerular Filtration Rates in Children. Clin Chem 2002;48:729-736.

18 Mazzachi BC, Peake MJ, Ehrhardt V. Reference Range and Method Comparison Studies for Enzymatic and Jaffé Creatinine Assays in Plasma and Serum and Early Morning Urine. Clin Lab 2000;53-55.

19 Schlebusch H, Liappis N, Klein G Creatinine and ultrasensitive CRP: Reference Intervals from Infancy to Childhood. Clin Chem Lab Med 2001;39 Special Supplement PO-T042;1-448.

20 Junge W, Wilke B, Halabi A, et al. Determination of reference intervals for serum creatinine, creatinine excretion and creatinine clearance with an enzymatic and a modified Jaffé method. Clin Chim Acta 2004;344:137-148.

21 Bablok W, Passing H, Bender R, et al. A general regression procedure for method transformation. Application of linear regression procedures for method comparison studies in clinical chemistry, Part III. J Clin Chem Clin Biochem 1988 Nov;26(11):783-790.

A point (period/stop) is always used in this Method Sheet as the decimal separator to mark the border between the integral and the fractional parts of a decimal numeral. Separators for thousands are not used.

Any serious incident that has occurred in relation to the device shall be reported to the manufacturer and the competent authority of the Member State in which the user and/or the patient is established.

Symbols

Roche Diagnostics uses the following symbols and signs in addition to those listed in the ISO 15223-1 standard (for USA: see dialog.roche.com for definition of symbols used):

CONTENT	Contents of kit

→	Volume after reconstitution or mixing
GTIN	Global Trade Item Number

FOR US CUSTOMERS ONLY: LIMITED WARRANTY

Roche Diagnostics warrants that this product will meet the specifications stated in the labeling when used in accordance with such labeling and will be free from defects in material and workmanship until the expiration date printed on the label. THIS LIMITED WARRANTY IS IN LIEU OF ANY OTHER WARRANTY, EXPRESS OR IMPLIED, INCLUDING ANY IMPLIED WARRANTY OF MERCHANTABILITY OR FITNESS FOR PARTICULAR PURPOSE. IN NO EVENT SHALL ROCHE DIAGNOSTICS BE LIABLE FOR INCIDENTAL, INDIRECT, SPECIAL OR CONSEQUENTIAL DAMAGES.

COBAS, COBAS C, COBAS INTEGRA, PRECINORM, PRECIPATH and PRECICONTROL are trademarks of Roche.

All other product names and trademarks are the property of their respective owners.

Additions, deletions or changes are indicated by a change bar in the margin.

© 2021, Roche Diagnostics

CE 0123

Roche Diagnostics GmbH, Sandhofer Strasse 116, D-68305 Mannheim
www.roche.com
+800 5505 6606

Roche

Distribution in USA by:
Roche Diagnostics, Indianapolis, IN
US Customer Technical Support 1-800-428-2336
